# Non-invasive Assessment of Liver Fibrosis Using Shear Wave Elastography in Patients With Type 2 Diabetes Mellitus Having Non-alcoholic Fatty Liver Disease

**DOI:** 10.7759/cureus.72471

**Published:** 2024-10-27

**Authors:** Deepthi Arun Kumar, Senthil Kumar, Revathi Rajagopal, Ragitha Ramesh, Manoj M

**Affiliations:** 1 Radiodiagnosis, SRM Medical College Hospital and Research Centre, Chengalpattu, IND

**Keywords:** 2d shear wave elastography, bmi, hba1c, lipid profile, liver fibrosis, liver stiffness, non-alcoholic fatty liver disease, type 2 diabetes mellitus, ultrasound

## Abstract

Background

Type 2 diabetes mellitus (T2DM) and non-alcoholic fatty liver disease (NAFLD) frequently coexist due to overlapping risk factors such as metabolic syndrome and obesity. T2DM exacerbates the progression of NAFLD, increasing the risk of cirrhosis and hepatocellular carcinoma. Thus, early detection of liver fibrosis is crucial to prevent severe liver disease. A 2D shear wave elastography (2D SWE) has emerged as a reliable non-invasive method for assessing liver stiffness, potentially reducing the need for liver biopsies and facilitating prompt treatment interventions.

Methods

This cross-sectional study, conducted over 18 months, included 100 T2DM and NAFLD patients from the Medicine and Diabetes Outpatient Department at SRM Medical College Hospital and Research Centre, Chengalpattu, India. Participants underwent gray-scale ultrasound to classify fatty liver (Grades I, II, and III) and 2D SWE to evaluate liver stiffness. Additional data on fasting and postprandial blood glucose, glycosylated hemoglobin (HbA1c), lipid profiles, liver function tests, and body mass index (BMI) were collected. Statistical analysis was performed using IBM SPSS Statistics for Windows, Version 21 (Released 2012; IBM Corp., Armonk, New York, United States).

Results

The mean age of participants was 47.9 years, with 61% being male. Fatty liver Grades I, II, and III were present in 47%, 41%, and 12% of patients, respectively. SWE results showed that 30% had stiffness values <5 kPa, 53% had values between 5.1-9 kPa, 16% had values between 9.1-13 kPa, and 1% had values >13 kPa. Liver size increased significantly with fatty liver grade (p=0.029). HbA1c levels and blood glucose levels were significantly correlated with fatty liver grades (p<0.0001). Triglyceride levels were higher with increasing fatty liver grades (p<0.0001). A significant correlation was found between gamma-glutamyl transferase (GGT) levels and SWE values (p=0.04). In the lipid profile, significant correlations were noted between SWE values and triglycerides (p=0.005), cholesterol (p=0.026), and very-low-density lipoprotein (VLDL) (p=0.131). Higher levels of HbA1c, fasting blood sugar, and postprandial blood sugar were also significantly correlated with SWE values (p<0.0001). Increasing grades of hepatic steatosis significantly correlated with SWE values (p<0.0001). BMI positively correlated with SWE values (r=0.321, p=0.001).

Conclusion

This study highlights the prevalence of advanced liver stiffness in patients with T2DM and NAFLD, which correlates significantly with higher grades of fatty liver, elevated HbA1c, blood sugar levels, and abnormal lipid profiles. SWE is a valuable tool for assessing liver stiffness and guiding the management of NAFLD in patients with T2DM.

## Introduction

Non-alcoholic fatty liver disease (NAFLD) is a condition of current global concern and has become the most prevalent chronic liver disease due to lifestyle changes and calorie-dense dietary habits of the general population. It is particularly notable in the South Asian population, with India bearing a prevalence ranging from approximately 9% to 32% [1]. It is often underestimated as a disease as it is mostly asymptomatic.

NAFLD encompasses a range of liver conditions characterized by lipid accumulation in hepatocytes, leading to hepatocyte injury, inflammation, and varying levels of fibrosis, without secondary causes such as alcohol use or viral hepatitis [2]. The disease is clinically heterogeneous; while many patients have isolated steatosis (non-alcoholic fatty liver, or NAFL), others develop non-alcoholic steatohepatitis (NASH), which can progress to advanced fibrosis and cirrhosis if untreated. NAFLD also increases the risk of cardiovascular diseases and liver complications, such as cirrhosis and hepatocellular carcinoma [3,4]. Early intervention through lifestyle modifications like diet, exercise, and weight management plays a crucial role in managing disease progression [5].

Type 2 diabetes mellitus (T2DM) frequently coexists with NAFLD, exacerbating its progression. Obesity and insulin resistance are closely linked to NAFLD, but patients with NAFLD face nearly double the risk of developing T2DM, independent of other metabolic risk factors [6]. This risk increases with the severity of NAFLD, and the prevalence of NAFLD among T2DM patients ranges from 34% to 94% globally [7]. Liver biopsy remains the gold standard for assessing fibrosis, but its invasiveness and sampling variability limit its use as a screening tool [8]. Consequently, non-invasive biochemical and radiological methods have been developed to evaluate liver fibrosis in NAFLD patients.

Elastography serves as a non-invasive modality for assessing liver fibrosis, with new methods including transient elastography, shear wave elastography, supersonic shear wave elastography, real-time elastography, and MRI elastography [9]. Among these, transient elastography and shear wave elastography (SWE) are widely accepted and accessible. While magnetic resonance elastography (MRE) is more precise than ultrasound-based elastography for detecting and staging liver fibrosis, it is also more expensive and less accessible [10].

Timely diagnosis and management are crucial to preventing cirrhosis since NAFLD affects about 70% of T2DM patients and is a feature of metabolic syndrome. With the increasing prevalence of NAFLD and variable diagnostic accuracy of 2D shear wave elastography, there is a pressing need for reliable, non-invasive diagnostic techniques. Shear wave elastography is preferred for its cost-effectiveness, non-invasiveness, real-time imaging capabilities, and ability to adjust the region of interest (ROI) of tissue, making it a valuable tool for assessing liver fibrosis and its progression [11]. This study was designed to address this need. Our study aims to evaluate the effectiveness and reliability of shear wave elastography for diagnosing liver fibrosis in patients with T2DM and NAFLD.

## Materials and methods

Study design and setting

This cross-sectional study was conducted at SRM Medical College Hospital and Research Centre, Kattankulathur, Chengalpattu, over an 18-month period from December 2022 to June 2024. A total of 100 patients were voluntarily enrolled. Ethical approval was obtained from the Institutional Ethical Committee for Students, with the reference number SRMIEC-ST0722-21. Informed consent was obtained from all study participants.

Inclusion and exclusion criteria

Inclusion criteria encompassed patients with type 2 diabetes mellitus according to WHO criteria and those with hepatic steatosis confirmed by B-mode ultrasound. Exclusion criteria included non-diabetic patients, individuals with alcoholic fatty liver disease, focal liver lesions, or other liver conditions, pregnant subjects, and those under 18 years of age. 

Methodology

Patients with T2DM who were suspected of having non-alcoholic fatty liver disease (NAFLD) underwent a gray-scale ultrasound to grade fatty liver into three categories: Grade I: increased liver echogenicity with clear visibility of the diaphragm and intrahepatic vessel borders; Grade II: increased echogenicity obscuring the walls of the portal vein and its branches; Grade III: high echogenicity that obscures the diaphragmatic outline.

Additionally, data on fasting and postprandial blood sugars, glycosylated hemoglobin (HbA1c), lipid profile, body mass index (BMI), liver function tests, and viral markers were collected. A 2D shear wave elastography (2D SWE) was performed using the Philips Affinity 70 Ultrasound System with Philips ElastQ imaging (Koninklijke Philips N.V., Amsterdam, Netherlands).

Technique

**Figure 1:**
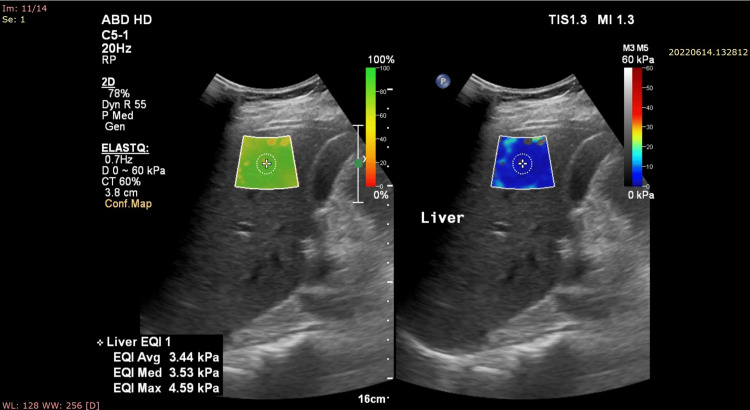
Showing a 2D SWE image of the liver in a 35-year-old female subject with Grade I fatty liver. The median value is noted to be 3.53 kPa. The patient had normal blood sugar parameters, lipid profile, and liver function test values. 2D SWE: 2D shear wave elastography

**Figure 2:**
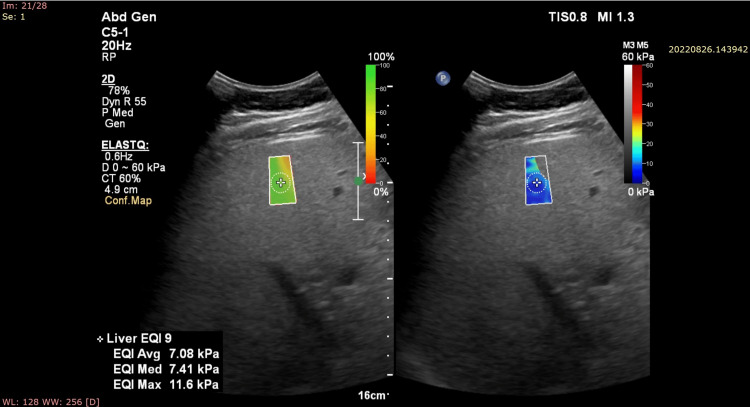
Showing a 2D SWE image of the liver in a 50-year-old male subject with Grade II fatty liver. The median value is noted to be 7.41 kPa. The patient had an HbA1c value of 7.9, fasting blood sugar of 201 mg/dL, and postprandial blood sugar of 260 mg/dL. The patient also had elevated cholesterol, triglyceride, and low-density lipoprotein (LDL) values, measuring 204 mg/dL, 189 mg/dL, and 130 mg/dL, respectively. The patient had normal liver function test values. 2D SWE: 2D shear wave elastography; HbA1c: glycosylated hemoglobin

The study participants underwent shear wave elastography after a minimum fasting period of six hours. The right lobe of the liver was visualized via intercostal spaces with the patient in a left lateral oblique position and the right arm extended. The transducer was placed parallel to the liver capsule, avoiding areas with significant vascular or biliary structures. The ROI box was positioned approximately 1.5-2 cm below the liver capsule to minimize artifacts. The area within the ROI box was verified with the confidence map, with green indicating high confidence. Patients were instructed to hold their breath for three to four seconds while images were captured to measure liver stiffness. Six measurements were taken, with the median value expressed in kPa (Figures 1, 2). The interquartile range (IQR)/median ratio was maintained below 30% for measurement quality (Appendix 1). Liver stiffness was interpreted based on Philips ElastQ shear wave elastography thresholds and the Society of Radiologists in Ultrasound guidelines for liver diseases (Table 1) [12].

**Table 1:** Recommendations for liver stiffness value interpretation according to the Consensus Update by the Society of Radiologists in Ultrasound (USA) on ultrasonic elastography for viral liver diseases and NAFLD. [12] NAFLD: non-alcoholic fatty liver disease

Liver Stiffness Value	Recommendation
Values equal to or less than 5 kPa (1.3 m/sec)	High probability of being normal
Values less than 9 kPa (1.7m/s)	In the absence of other clinical signs, exclude compensated advanced chronic liver disease. If there are known clinical signs, further tests are required to confirm.
9 to 13 kPa (1.7- 2.1 m/s)	Suggestive of compensated advanced chronic liver disease, needs further tests for confirmation.
Values above 13 kPa (2.1 m/s)	Rules in compensated advanced chronic liver disease.
Values above 17 kPa (2.4 m/s)	Suggestive of significant portal hypertension.

Statistical analysis

Data were presented as means and standard deviations, and continuous variables were compared using the independent sample t-test. Significance was defined by P-values less than 0.05 using a two-tailed test. Data analysis was performed using IBM SPSS Statistics for Windows, Version 21 (Released 2012; IBM Corp., Armonk, New York, United States).

## Results

Demographics and baseline characteristics

The study comprised 100 subjects with type 2 diabetes mellitus (T2DM) and non-alcoholic fatty liver disease (NAFLD). The majority (38%) were aged 41-50 years, with a mean age of 47.9±9.6 years. The cohort was predominantly male (61%) compared to female (39%). B-mode ultrasound revealed that 47% had Grade I fatty liver, 41% had Grade II, and 12% had Grade III.

The 2D shear wave elastography (SWE) results showed that 53% of patients had SWE values between 5.1-9 kPa, 30% had values less than 5 kPa, 16% had values between 9.1-13 kPa, and 1% had values above 13 kPa (Table 2).

**Table 2:** Depicting the demographics and baseline characteristics of the study group. In our study cohort, the majority of patients 38% (N=38), were between 41 and 50 years of age. The gender distribution was 61% (N=61) male and 39% (N=39) female. Regarding the grading of fatty liver, 47% (N=47) of the patients had Grade I fatty liver, 41% (N=41) had Grade II, and 12% (N=12) had Grade III. SWE: shear wave elastography

		Number of Patients (N)	Percentage (%)
Age group (in years)	<40	25	25.0%
	41-50	38	38.0%
	51-60	27	27.0%
	>61	10	10.0%
Gender	Female	39	39.0%
	Male	61	61.0%
USG Grade	1	47	47.0%
	2	41	41.0%
	3	12	12.0%
kPa SWE	<5	30	30.0%
	5.1-9	53	53.0%
	9.1-13	16	16.0%
	>13.1	1	1.0%

Correlation of liver size, liver function tests, blood sugar parameters, and lipid profile with steatosis grades

Mean liver sizes were 13.08 cm for Grade I, 13.4 cm for Grade II, and 14.17 cm for Grade III fatty liver (Table 3), with a significant correlation between liver size and fatty liver grades (p=0.029). Liver function tests (aspartate aminotransferase (AST), alanine transaminase (ALT), gamma-glutamyl transferase (GGT), alkaline phosphatase (ALP)) did not show significant correlations with fatty liver grades. However, HbA1c, fasting blood sugar (FBS), and postprandial blood sugar (PPBS) levels showed significant statistical correlations (p<0.0001). Lipid profile analysis indicated significant correlations between triglycerides and fatty liver grades (p<0.0001) and between cholesterol and fatty liver grades (p=0.046) (Table 3).

**Table 3:** Depicting correlation of liver size, liver function tests, blood sugar parameters, and lipid profile with steatosis grades. Liver size, liver function test, blood sugar, and lipid profile values are presented as mean ± standard deviation. A p-value <0.05 was considered statistically significant in our study. AST: aspartate aminotransferase; ALT: alanine transaminase; GGT: gamma-glutamyl transferase; ALP: alkaline phosphatase; FBS: fasting blood sugar; PPBS: postprandial blood sugar; HDL: high-density lipoprotein; LDL: low-density lipoprotein; VLDL: very-low-density lipoprotein; HbA1C: glycosylated hemoglobin

		USG Grade						P-value
		1		2		3		
		Mean	Standard Deviation	Mean	Standard Deviation	Mean	Standard Deviation	
Liver size		13.08	0.99	13.40	1.27	14.17	1.98	0.029
Liver function test (U/L)	AST	26.62	7.69	30.41	19.12	29.75	11.35	0.423
	ALT	30.43	10.80	32.37	17.69	34.17	11.91	0.66
	GGT	28.00	10.40	33.93	16.70	34.00	14.54	0.104
	ALP	84.55	18.04	89.10	14.92	80.00	20.49	0.213
Blood sugar values (mg/dL)	HbA1c	5.91	1.21	7.87	1.88	7.73	1.85	<0.0001
	FBS	111.49	33.74	163.22	55.84	178.50	84.37	<0.0001
	PPBS	157.26	64.56	235.07	81.61	268.67	121.92	<0.0001
Lipid profile (mg/dL)	Cholesterol	168.23	47.27	190.41	45.81	195.67	47.81	0.046
	Triglycerides	126.83	92.51	188.83	120.86	275.83	149.86	<0.0001
	HDL	48.06	13.24	44.85	8.62	46.25	7.72	0.309
	LDL	119.81	29.04	129.71	42.08	131.42	41.74	0.371
	VLDL	28.96	16.07	33.93	16.23	34.42	12.15	0.277

Correlation of liver size, steatosis grades, liver function tests, blood sugar parameters, lipid profile, and BMI with SWE values

SWE values did not show a significant correlation with liver size. Among liver function tests, only GGT levels correlated significantly with SWE values (p=0.04). Significant correlations were observed between SWE values and blood sugar parameters, including fasting blood sugar (FBS) and postprandial blood sugar (PPBS) (p<0.0001), as well as HbA1c levels (p=0.04). The lipid profile revealed significant correlations with SWE values for cholesterol (p=0.026), triglycerides (p=0.005), and very-low-density lipoprotein (VLDL) levels (p=0.131) (Table 4).

**Table 4:** Depicting correlation of liver size, liver function tests, blood sugar parameters, and lipid profile with SWE values. Liver size, liver function test, blood sugar, and lipid profile values are presented as mean ± standard deviation. A p-value <0.05 was considered statistically significant in our study. AST: aspartate aminotransferase; ALT: alanine transaminase; GGT: gamma-glutamyl transferase; ALP: alkaline phosphatase; FBS: fasting blood sugar; PPBS: postprandial blood sugar; HDL: high-density lipoprotein; LDL: low-density lipoprotein; VLDL: very-low-density lipoprotein; SWE: shear wave elastography; HbA1c: glycosylated hemoglobin

		kPa SWE								P-value
		<5		5.1-9		9.1-13		>13.1		
		Mean	Standard Deviation	Mean	Standard Deviation	Mean	Standard Deviation	Mean	Standard Deviation	
Liver size (cm)		13.06	1.05	13.41	1.27	13.60	1.72	14.00	n/a	0.48
Liver function test (U/L)	AST	27.23	8.07	29.06	16.76	29.88	12.73	20.00	n/a	0.838
	ALT	32.07	10.69	31.77	15.61	30.94	15.46	26.00	n/a	0.973
	GGT	27.00	10.24	31.11	12.17	39.31	21.60	27.00	n/a	0.04
	ALP	87.43	16.61	83.79	18.17	90.50	15.25	75.00	n/a	0.469
Blood sugar values (mg/dL)	HbA1c	5.86	1.31	7.23	1.95	7.79	1.48	9.70	n/a	<0.0001
	FBS	110.40	39.57	146.57	54.10	168.13	67.23	304.00	n/a	<0.0001
	PPBS	158.10	85.47	206.42	76.32	260.25	98.13	406.00	n/a	<0.0001
Lipid profile (mg/dL)	Cholesterol	171.00	52.78	176.57	42.46	213.00	45.12	166.00	n/a	0.026
	Triglycerides	107.87	82.07	188.60	129.37	224.88	119.25	183.00	n/a	0.005
	HDL	47.23	12.26	43.91	11.55	41.25	8.70	37.00	n/a	0.313
	LDL	118.83	39.32	124.45	26.98	140.50	53.97	117.00	n/a	0.285
	VLDL	24.73	15.95	33.89	15.58	36.88	12.93	37.00	n/a	0.131

Regarding the relationship between SWE values and fatty liver grades, 57.4% (N=27) of patients with Grade I fatty liver had SWE values <5 kPa. Among patients with Grade II fatty liver, 75.6% (N=31) had SWE values between 5.1 and 9 kPa, while 75% (N=9) of those with Grade III fatty liver had SWE values between 9.1 and 13 kPa. These findings indicate that the majority of patients with more advanced grades of fatty liver exhibited proportionately higher liver stiffness measurements. A statistically significant correlation was observed between increasing SWE values and advancing fatty liver grades (p<0.0001).

A positive correlation was noted between BMI and SWE values (r=0.321, p=0.001), indicating that as BMI increases, there is a corresponding increase in stiffness measured by kPa SWE.

## Discussion

Non-alcoholic fatty liver disease (NAFLD) can cause chronic liver damage, ultimately leading to fibrosis and increased liver stiffness. Liver elastography serves as a valuable diagnostic modality for the non-invasive assessment of liver pathology, including fibrosis. Over the past two decades, elastography has become an established method for evaluating liver stiffness. Among the available techniques, two-dimensional (2D) ultrasound-based elastography is particularly advantageous due to its ease of use, accessibility in outpatient settings, and ability to provide real-time imaging for the detection of liver fibrosis [13]. This study aimed to quantify liver stiffness using 2D shear wave elastography in patients with NAFLD and type 2 diabetes mellitus (T2DM) and to explore the relationship between liver stiffness measurements, sonographic grading, and various biochemical parameters.

In our study of 100 patients, the majority (38%) were aged 41-50 years, with a mean age of 47.9±9.6 years. The age distribution highlights a middle-aged predominance, emphasizing the need for targeted early intervention in this demographic. Regarding gender distribution, our study found a male predominance (61%) compared to females (39%), which aligns with other studies showing a higher prevalence of NAFLD in men. Yilmaz et al. observed 51% of males with NAFLD and T2DM in their study [14].

Sonographic grading revealed 47% of patients with Grade I fatty liver, 41% with Grade II, and 12% with Grade III among our study subjects. Shear wave elastography (SWE) revealed that 53% of patients had values between 5.1 and 9 kPa, indicating mild to moderate liver stiffness, while 30% had values below 5 kPa, and 16% had values between 9.1 and 13 kPa, with only 1% exceeding 13 kPa. This distribution suggests that most patients have mild to moderate liver fibrosis, with a small proportion showing more severe stiffness. Meyer et al. reported that 54% of T2DM patients had SWE values <7 kPa, and Riestra-Candelaria et al. observed that most patients with T2DM had SWE values between 5.48 and 8.29 kPa, which aligns closely with our findings [15,16]. Chimoriya et al. also conducted 2D shear wave elastography on patients with Class 3 obesity presenting with either NAFLD, T2DM, or both and observed that 79.7% of patients had values <9.4 kPa [17]. Our study’s findings are consistent with these studies, reinforcing the utility of SWE in differentiating liver fibrosis severity (Table 5).

**Table 5:** Metabolic parameters, liver size, and grades of hepatic steatosis (correlation of our study with previously published studies regarding SWE in NAFLD patients). AST: aspartate aminotransferase; ALT: alanine transaminase; GGT: gamma-glutamyl transferase; HDL: high-density lipoprotein; LDL: low-density lipoprotein; TG: triglycerides; SWE: shear wave elastography; NAFLD: non-alcoholic fatty liver disease; BMI: body mass index; HbA1c: glycosylated hemoglobin

Study	Mean Age	Gender Distribution	Liver Size	Grades of Steatosis	Liver Function Test	Lipid Profile	Fasting Blood Sugar	HbA1c	BMI
Our study	47.9 ± 9.6	61% male, 39% female	Not significant (p 0.048)	Significant (p<0.0001)	GGT significant (p 0.04)	Cholesterol (p 0.026) and TG (p 0.005) significant	Significant (p<0.0001)	Significant (p<0.0001)	Significant (p 0.001)
Yilmaz et al., 2020 [14]	55	51% male, 49% female	Not significant	Not significant	ALT, AST significant (p<0.001)	TG significant (p<0.001)	Not available	Not available	Not significant
Meyer et al., 2021 [15]	63	55% male, 45% female	Not available	Not available	Not available	Not significant	Not available	Not significant	Significant (p= 0.003)
Riestra-Candelaria et al., 2020 [16]	Not available	Not available	Not significant (p 0.24)	Not available	Not significant	Not available	Not available	Significant (p<0.001)	Not available
Miyoshi et al., 2021 [18]	63.4±12.2	57% male, 43% female	Not available	Significant (p<0.001)	Total bilirubin, AST, ALT significant (p 0.006, 0.04, 0.018)	Cholesterol, TG, HDL significant (p 0.041, 0.005, <0.001)	Significant (p=0.046)	Not significant (p 0.05)	Significant (p <0.001)
Shaheen et al., 2020 [19]	55	46.3% male, 53.7% female	Not available	Not available	ALT, AST, GGT significant (p<0.001)	Cholesterol and LDL significant (p <0.001)	Significant (p 0.002)	Significant (p<0.0001)	Significant (p <0.001)
Jamialahmadi et al., 2019 [20]	38.5±11.1	20% male, 80% female	Not available	Significant (p<0.0001)	Not available	Not available	Not available	Not available	Significant (p 0.004)

In examining the relationship between liver size and grades of fatty liver, our study found that liver size increased with the severity of fatty liver. Statistical analysis indicated a significant correlation between liver size and fatty liver grade (p=0.029). Meyer et al. and Miyoshi et al. both found that increased liver size is associated with higher grades of fatty liver and a higher risk of developing NAFLD, corroborating the results of our study [15,18].

The correlation between SWE values and liver size in our study was not significant (p=0.48), suggesting that liver size alone may not be a reliable indicator of liver stiffness. A study by Yilmaz et al. on NAFLD subjects with T2DM similarly found no significant correlation between increasing liver sizes and shear wave velocities [14].

Liver function tests in our study did not show a significant correlation with SWE values, except for gamma-glutamyl transferase (GGT) levels, which exhibited a significant correlation (p=0.04). This finding aligns with research by Shaheen et al., who found significant associations between GGT, AST, and ALT with liver fibrosis [19]. However, no correlation was noted between AST, ALT, and increasing liver stiffness in our study.

Significant correlations were found between lipid profile parameters and SWE values, particularly for cholesterol (p=0.026) and triglycerides (p=0.005). Patients with higher SWE values had notably higher triglyceride levels. These results are consistent with Yilmaz et al., who demonstrated a link between triglycerides (p<0.001) and liver stiffness, reinforcing the association between dyslipidemia and increased liver fibrosis [14]. Additionally, Miyoshi et al. reported significant relationships between cholesterol (p=0.026), triglycerides (p=0.005), and liver stiffness in patients with abdominal obesity [18]. Shaheen et al. also found a significant correlation with cholesterol (p<0.001) [19].

We found a significant correlation between SWE values and HbA1c, fasting blood sugar (FBS), and postprandial blood sugar (PPBS) levels. This aligns with findings from Shaheen et al., who also observed significant correlations between HbA1c and blood sugar levels with liver stiffness, highlighting the association between poor glycemic control and increased liver stiffness [19].

Our study identified a significant positive correlation between body mass index (BMI) and SWE values (Pearson correlation=0.321, p=0.001), suggesting that higher BMI is associated with increased liver stiffness. This finding aligns with Meyer et al., who reported a correlation between BMI and liver fibrosis stages in T2DM patients (p=0.003) [15]. Shaheen et al. (p<0.001) also observed similar findings [19]. Jamialahmadi et al. conducted a study on severely obese candidates for bariatric surgery and observed a significant link between BMI and SWE values (p<0.004), with elevated BMI predicting higher SWE values [20] (Table 5). Overall, our results corroborate the connection between higher BMI and increased liver stiffness.

Limitations

Our study had limitations that need addressing. Firstly, the lack of liver biopsies prevented accurate staging of fibrosis in patients with non-alcoholic fatty liver disease (NAFLD). Additionally, including patients with altered liver function tests (LFTs) could confound the results, as elevated LFTs may independently affect liver stiffness measurements from 2D shear wave elastography (SWE), potentially masking true fibrosis levels. Lastly, the study's single-center design and limited sample size of 100 subjects restrict the generalizability of the findings. Future research should involve multiple centers and larger, more diverse populations to better assess the diagnostic performance and reliability of SWE.

## Conclusions

Our study revealed a high prevalence of fatty liver among patients with type 2 diabetes mellitus (T2DM), predominantly presenting as Grades I and II. Two-dimensional shear wave elastography (2D SWE) demonstrated effectiveness in assessing liver stiffness, which is crucial for the diagnosis and management of liver conditions. A significant increase in liver size was observed with the severity of fatty liver, indicating a direct correlation between liver enlargement and fatty liver grades.

Moreover, significant associations were identified between metabolic factors and the severity of fatty liver; elevated HbA1c, as well as fasting and postprandial blood sugar levels, were linked to higher fatty liver grades and increased liver stiffness measured by SWE. Additionally, higher levels of triglycerides and cholesterol were associated with greater liver stiffness.

These findings emphasize the necessity for comprehensive metabolic control and regular monitoring of liver function in T2DM patients with NAFLD. The study particularly highlights the utility of SWE as a non-invasive method for assessing liver fibrosis, suggesting its pivotal role in the early detection and management of liver disease in diabetic patients.

## Figures and Tables

**Figure 3:**
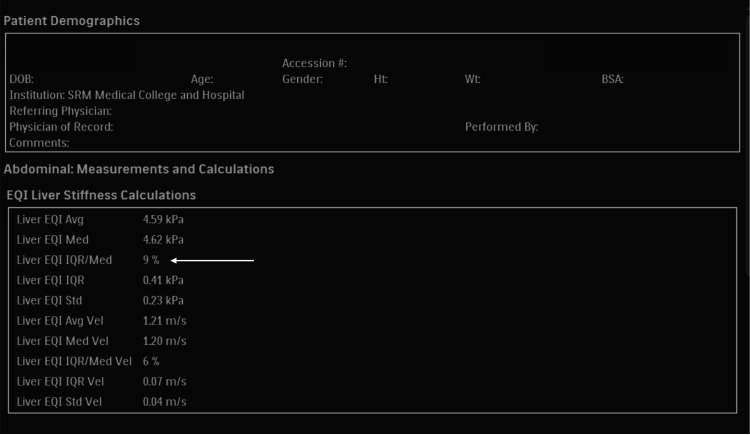
Showing the IQR/median ratio (white arrow) maintained below 30% to maintain good quality of measurements. This 2D SWE study was conducted on a 35-year-old male subject with Grade I fatty liver. The median value was noted to be 4.62 kPa after obtaining six readings. The generated report shows the SWE measurements in both kPA and m/s. The IQR/median (Med) velocity for m/s has no clinical importance. SWE: shear wave elastography

## Appendices

Appendix 1 
